# Designing a novel tetradentate polyoxometalate eco-catalyst for the synthesis of β-aminocyclohexanone derivatives in water†

**DOI:** 10.1039/c8ra08259f

**Published:** 2018-12-03

**Authors:** Roya Mozafari, Fariba Heidarizadeh, Maedeh Azaroon

**Affiliations:** Chemistry Department, College of Science, Shahid Chamran University Ahvaz 61357-4-3169 Iran heidarizadeh@scu.ac.ir (+98) 61 33738044 (+98) 6135743169

## Abstract

The synthesis of a series of known β-aminocyclohexanones has been accomplished using pentaerythrityl tetramethyl imidazolium phosphotungstate (C(MIM-PTA)_4_) as a new tetradentate acidic catalyst. It was prepared *via* condensation of pentaerythrityl tetrabromide with methyl imidazole. Then, bulky anion H_2_PW_12_O_40_^1−^ was substituted with Br^−^ in the structure. This tetradentate catalyst provides designable cations and anions. Anions have two types of acids, acidic protons, and metals with Lewis acidity. In order to test the efficient catalytic behavior of the tetradentate catalyst, a controlled reaction was performed using benzaldehyde, aniline and cyclohexanone. Imine from the condensation of benzaldehyde and aniline was observed in the absence of ionic catalyst instead of desired products. Thus, this reaction would be attractive because of the time, energy, and raw material saving considerations because of the absence of isolation of intermediates and stereospecificity. The catalyst shows high catalytic activity such that after four recycles the product was obtained with high yield and purity. This reaction was performed at room temperature. Although high temperature could improve the reaction rate, it contributes to side reactions and oxidation of aldehyde and amine. The catalyst was characterized by elemental analysis, FT-IR spectroscopy, ^1^H NMR, ^13^C NMR, and TGA.

## Abstract 2

The synthesis of a series of known β-aminocyclohexanones has been accomplished using pentaerythrityl tetramethyl imidazolium phosphotungstate (C(MIM-PTA)_4_) as a new tetradentate acidic catalyst.

## Graphical abstract

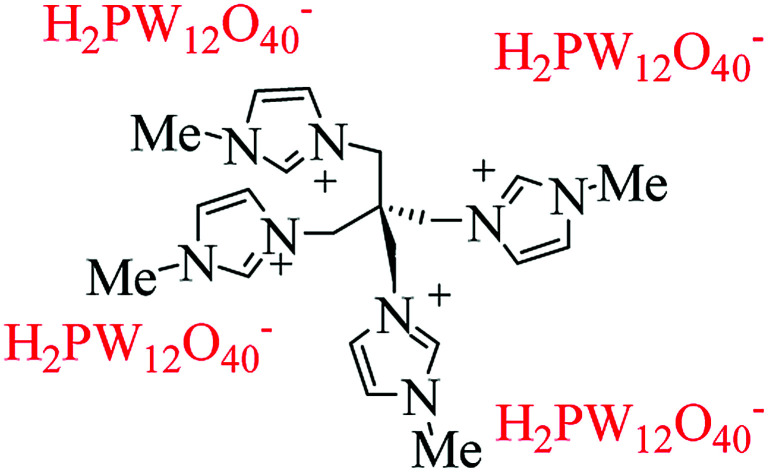

## 1. Introduction

The Mannich reaction is one of the most useful strategies for C–C bond formation in organic synthesis.^1,2^ It is a practical and efficient method for the construction of both allylic and benzylic amines, important targets that are found in many biologically active molecules and which are useful synthetic intermediates employed in the total syntheses of alkaloids and other complex molecules.^3–7^ This reaction is considered to be a very effective strategy for the synthesis of useful compounds as building blocks in pharmaceuticals and natural products such as antimicrobial,^8^ cytotoxic,^9^ anticancer^10^ and analgesic activity.^11,12^

Polyoxometalates (POMs), discrete anionic metal-oxo clusters, can be linked together through cationic moieties to build materials with incredible structural diversity which exhibit a wide variety of compositions and structural versatility, as well as important optical, catalytic, and magnetic properties.^13–18^ The POM compounds have been used as efficient catalysts in various chemical transformations due to their strong Brønsted acidity, thermal stabilities, lower corrosivity and higher catalytic activity.^19–23^

The general catalysts for standard Mannich reactions mainly involve various acids. Most of these strategies suffer from problems such as using corrosive reagents, expensive and large amounts of catalyst, long reaction times, harmful reaction media (fluorinated solvent), and low yields. In this regard, aromatic amines with *ortho*-substitution in most cases gave trace or even no products as a result of a large steric hindrance effect. From economic and environmental perspectives, the favored route is to utilize a one-pot three-component route incorporating a large spectrum of structural diversity.^24–37^

**Fig. 1:**
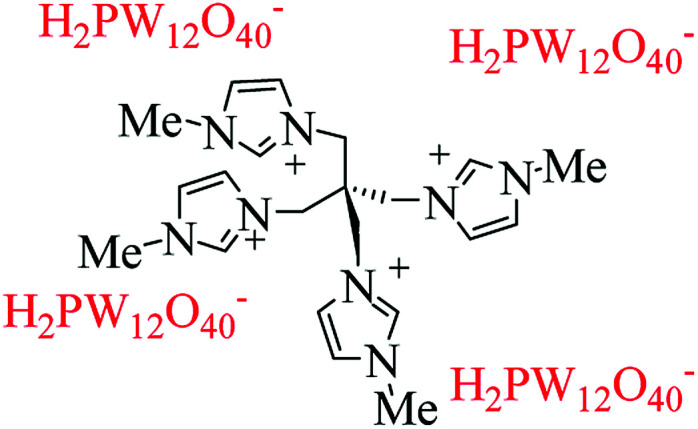
Tetradentate catalyst (pentaerythrityl tetramethylimidazolium phosphotungstate C(MIM-PTA)_4_).

Among the heteropolyacid (HPA) catalysts, H_3_PW_12_O_40_ (HPW) as a Brønsted acid has higher strength than H_2_SO_4_.^38^ Metal salts of PW_12_O_40_^3−^ potentially show the Lewis acidity originating from the metal cations as the electron pair acceptors as well as Brønsted acidity of protons.^39^ They have different hydrated structures depending upon the environment. Therefore, Lewis acidic HPAs could be introduced by modular grafting Lewis cations onto the phosphotungstic backbone. HPAs used in this research are the well known inorganic modifiers in view of their crystalline nature, high conductivity and thermally stable properties. In the present work, we describe the successful synthesis and use of a tetradentate polyoxometalate (Fig. 1) for the condensation of aldehyde, ketone and amine in the Mannich-type coupling. This designable catalyst has some important properties such as a heterogenic Brønsted and Lewis catalyst, the presence of oxygen as a base, and recyclability.

## 2. Experimental

### 2.1. Materials

Methylimidazole (99%), pentaerythrityl tetrabromide (98%), all solvents and other chemicals were purchased from Fluka and Merck companies and used without further purification. The purity determination of the products and reaction monitoring were accomplished by TLC on silica gel polygram SILG/UV 254 plates. Products were characterized by comparison of their physical and spectral data with those reported in the literatures.

### 2.2. Instrumentation

NMR spectra were taken using a BRUKER Advance 250 and 400 MHz NMR spectrometer and deuterated DMSO. FT-IR spectra of the powders were recorded utilizing BOMEM MB-Series 1998 FT-IR spectrometer. Mass spectra were obtained by a FINNIGAN-MAT 8430 mass spectrometer operating at an ionization potential of 70 eV. The TGA curve was recorded on a PC Luxx 409 under a nitrogen atmosphere at heating rates of 10 °C min^−1^.

### 2.3. Preparation of methyl pentaerythrityl tetramethylimidazolium phospho-tungstate (C(MIM-PTA)_4_)

A mixture of methylimidazole (8 mmol, 0.65 mL) and pentaerythrityl tetrabromide (0.77 g, 2 mmol) was heated and magnetically stirred at 60 °C for 48 h. Diethyl ether (15 mL) was added to the reaction mixture, after cooling to room temperature. At this stage, two-phase solutions appeared and the bottom phase was separated. Then, the ethereal part was evaporated and the white solid appeared. The obtained solid was washed three times with acetonitrile and the residue was dried at 80 °C for 2 h, giving tetramethyl imidazolium bromide C(MIM-Br)_4_ as a white powder (yield: 93%, melting point = 174 °C).

In the following step, we substituted Br^−^ by bulky anion PW_12_O_40_^3−^. This led to the formation of organic–inorganic hybrid solid material. The C(MIM-Br)_4_ and phosphotungstic acid (PTA) (H_3_PW_12_O_40_·*n*H_2_O) were taken in 1 : 4 mmol ratio to prepare one mmol of methyl pentaerythrityl tetramethylimidazolium phospho-tungstate. For this reason, (0.5 mmol, 0.351 g) of pentaerythrityl tetramethylimidazolium bromide was added drop wise to the solution containing (2 mmol, 5.6 g) g of PTA under constant stirring at room temperature. The white precipitate obtained was washed with distilled water and dried at 80 °C for overnight (yield: 94%).

### 2.4. A typical procedure for the preparation of β-aminocyclohexanones

Typically, to a mixture of aldehyde (1 mmol), amine (1 mmol), ketone (2 mmol) and H_2_O (5 mL) were added the catalyst (0.05 g) and was vigorously stirred at room temperature for the appropriate time. Completion of the reaction was monitored by TLC. Then, the reaction mixture was cooled to room temperature and diluted with hot ethanol (20 mL). The ethanolic solution might have been filtered to isolate the heterogeneous catalyst. The ethanol was then evaporated under reduced pressure to obtain the product. The white crystal was recrystallized with 10 mL ethanol or by thin-layer chromatography on 20 × 20 plates of silicagel 60 GF254 with *n*-hexane/EtOAc as eluent. Products are known compounds, and their NMR spectra are in accordance with those reported in the literature.

## 3. Results and discussion

Heteropoly acids are used as a homogeneous catalyst owing to their good solubility in various solvents. Homogeneous catalysts have some major disadvantages that limit the use of these compounds. They are stable only in relatively mild conditions that limit their uses and since the catalysts are dispersed in the reaction media, the separation process is difficult (or impossible) and expensive.

One of the general route to make heterogeneous polyoxometalate catalysts is by loading them in porous materials. This heterogeneous catalyst showed a high activity in many catalytic reactions, but they suffered aggregation and leaching of the catalyst due to the weak interaction with supports. Besides, these hydrophilic polyoxometalate catalysts are insoluble in apolar reaction systems. Therefore, designing a new class of heterogeneous catalyst by the pairing of a polyoxometalate anion with an appropriate cation would be a good candidate instead of porous support.

In this work, we report the synthesis of a versatile, efficient, and reusable heterogeneous pentaerythrityl tetramethyl imidazolium phosphotungstate as a tetradentate acidic catalyst and preparation of a series of β-aminocyclohexanones utilizing this new catalyst.

**Scheme 1:**
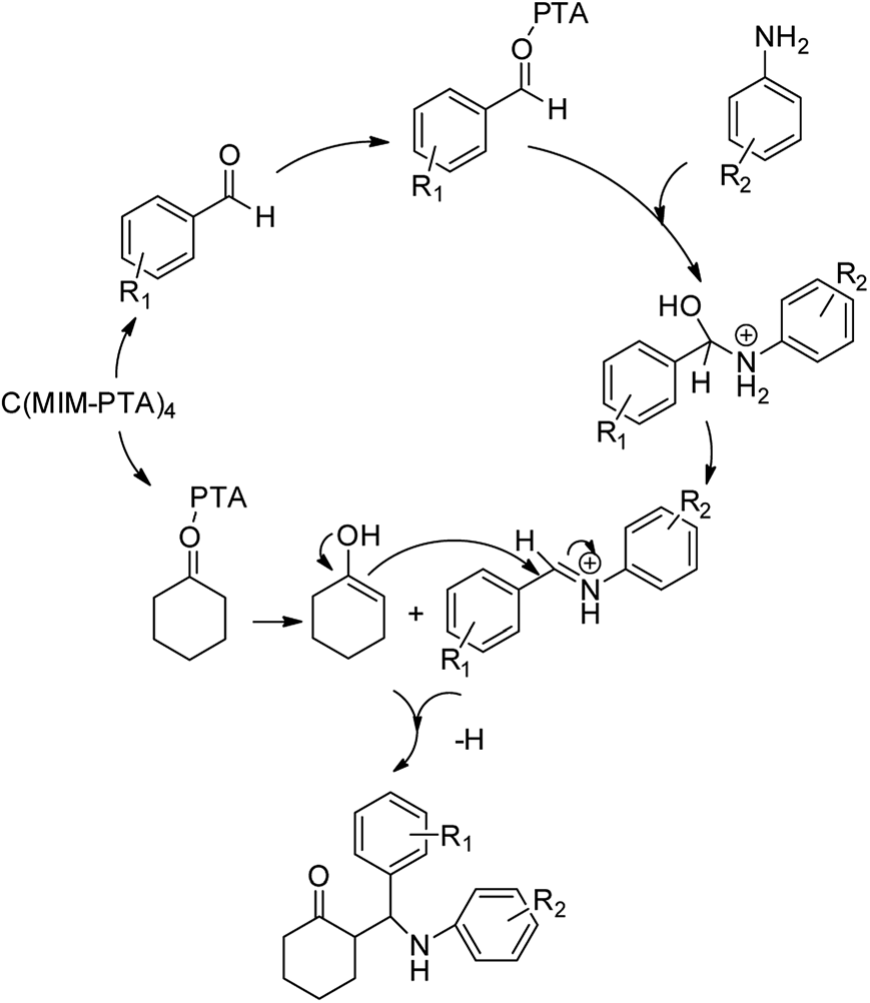
Proposed mechanistic pathway for the C(MIM-PTA)_4_ promoted synthesis of β-aminocyclohexanones.

To verify the efficiency of the catalyst, a controlled reaction was performed using benzaldehyde (1 mmol), aniline (1 mmol), and cyclohexanone (2 mmol) at room temperature. In the absence of the catalyst, imine from the condensation of benzaldehyde and aniline were observed (Scheme 1).

However, under the same condition, by employing 0.05 g of C (MIM-PTA)_4_, the reaction afforded expected products up to 95% yield within 1 h of reaction time without isolation of intermediates. Table 1 summarizes the results.

**Table 1:** The one-pot synthesis^a^ of β-aminocyclohexanones using C(MIM-PTA)_4_

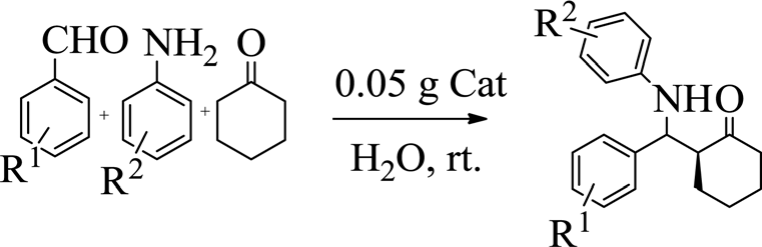						
Entry	Aldehyde	Aniline	Product	Time (min)	Yield^b^ (%)	Mp (°C) found (reported)
1	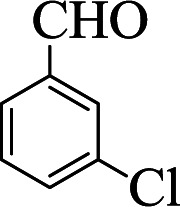	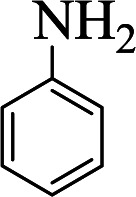	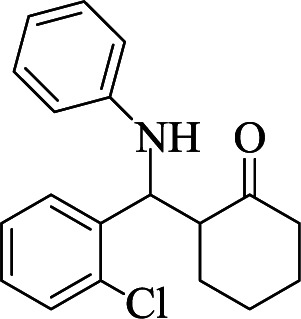	90	90	137–140 (137–139) ^40^
2	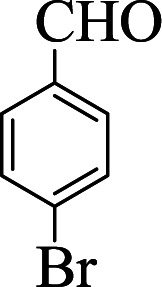	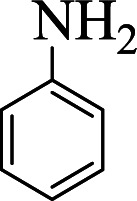	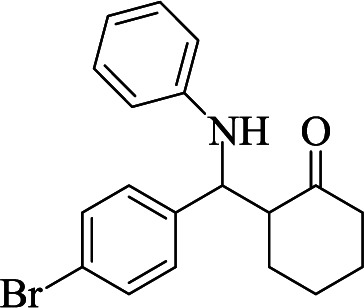	50	88	107–109 (109–110) ^42^
3	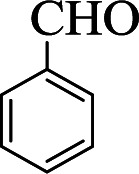	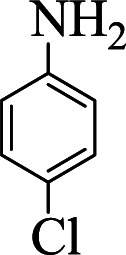	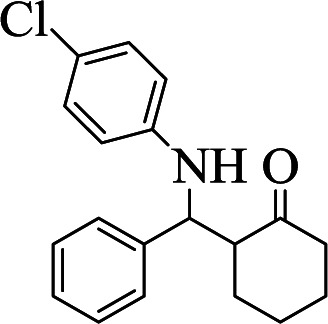	65	95	138–136 (137) ^43^
4	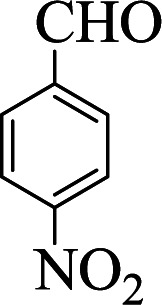	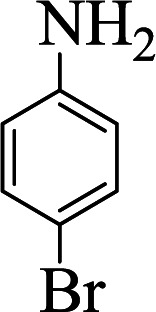	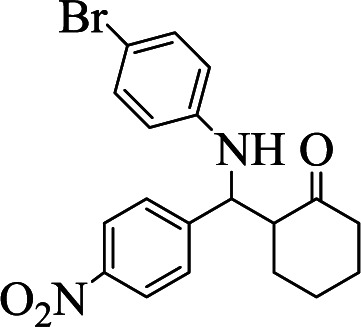	50	93	134–135 (134–136) ^43^
5	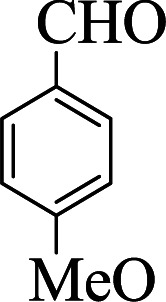	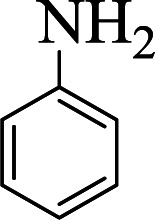	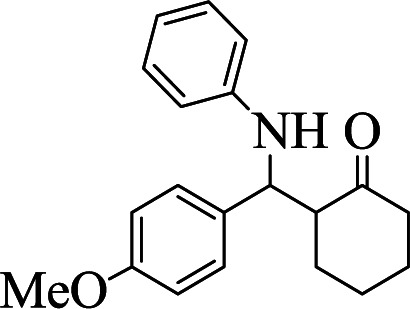	90	84	122–125 (120–122) ^43^
6	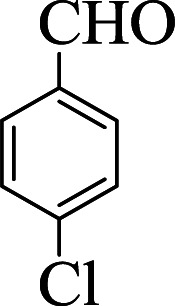	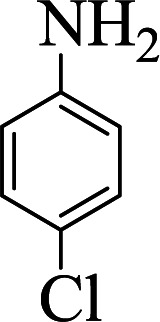	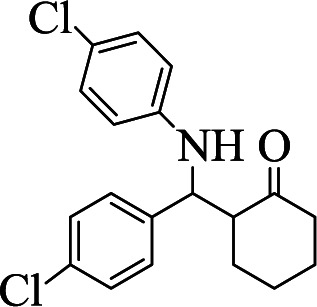	55	86	260–264 (262–263) ^40^
7	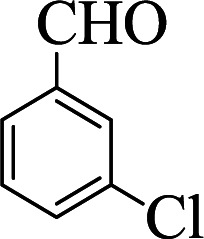	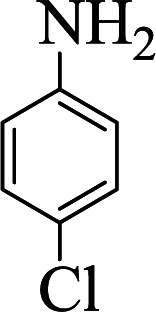	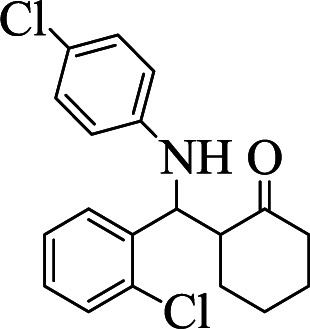	60	89	210–211 (211–213) ^44^
8	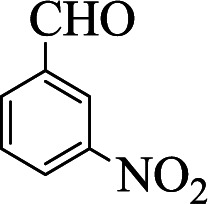	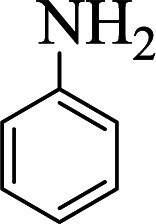	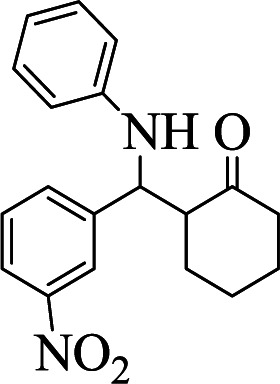	45	92	159–161 (161–163) ^45^
9	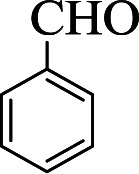	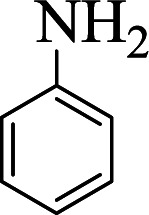	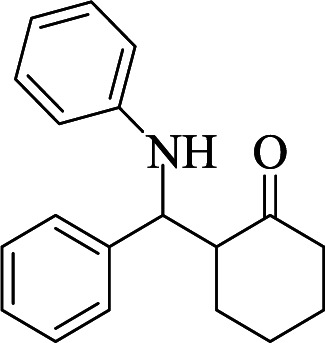	60	95	127–128 (129) ^46^

a Reaction conditions: aldehyde (1 mmol), cyclohexanone (2 mmol) and aniline (1 mmol), water (5 mL), C (MIM-PTA)_4_ (0.05 g) was vigorously stirred at room temperature for the appropriate time. Completion of the reaction was monitored by TLC.

b The yields refer to isolated products.

The solvent-free condition or using H_2_O with 0.05 g catalyst at room temperature is apparently the best condition for these reactions. The use of other organic solvents such as CH_2_Cl_2_, CH_3_CO_2_Et, CH_3_CN, and EtOH afforded the desired product in lower yields. The optimum reaction temperature for this catalyst was the room temperature. Although higher temperatures could improve the reaction rate, they contribute to side reactions. We observed that the Mannich reaction was very temperature-sensitive.

Stereoselectivity of β-aminocyclohexanones was determined by ^1^H NMR spectroscopy found that anti-isomer was the only product of the reaction in all cases. It is notable that the *ortho*-substituted aromatic amines were produced scarcely due to the large steric hindrance.

The hybrid catalysts are completely characterized by the aid of FT-IR spectroscopy, ^1^H NMR, ^13^C NMR, elemental analysis, and TGA.

**Fig. 2:**
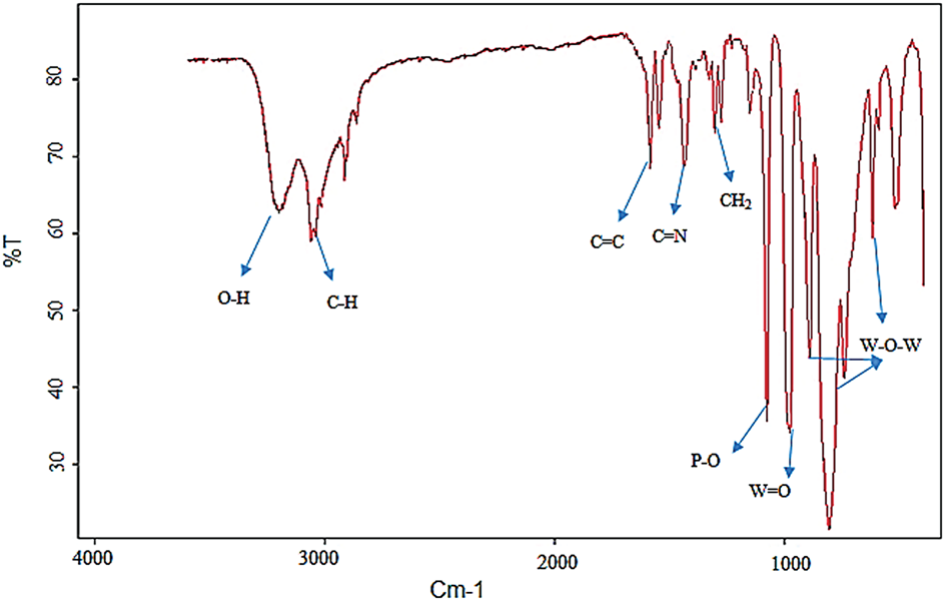
FT-IR spectra of pentaerythrityl tetramethylimidazolium phosphotungstate.

To characterize the catalyst, FT-IR spectroscopy was utilized (Fig. 2). The adsorption band at 1080 cm^−1^ can be attributed to the stretching of the P–O bond. The peaks at 981 and 806–892 cm^−1^ are due to the bending vibration mode of W=O and W–O–W bonds. The peaks at 3320 cm^−1^ and 1255 cm^−1^ were assigned to stretching vibration of CH bonds in methylene groups. The peaks at 1560 and 1670 cm^−1^ were assigned to imine and C=C bands in the catalyst, and the band in 3310 cm^−1^ arises from OH.

**Fig. 3:**
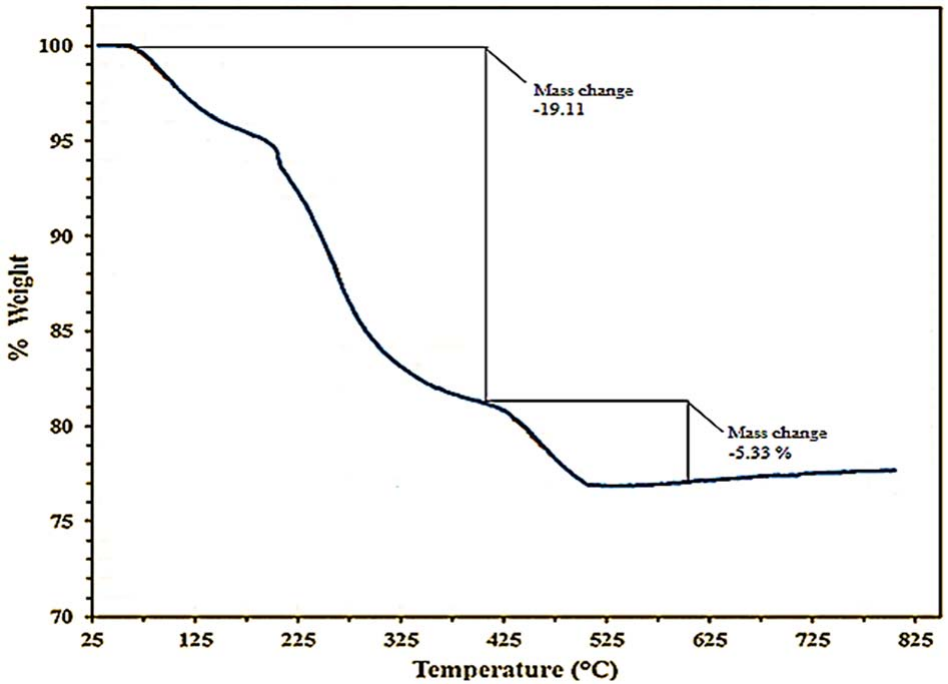
TGA curve of C(MIM-PTA)_4_.

The stability of the catalyst was examined by thermo-gravimetric analysis, TGA. The curve shows that three distinct steps of weight loss in the combined TGA curve. The first step occurred over the range 100–250 °C due to the loss of absorbed water. The second step had a weight loss over the range 225–425 °C and might be due to the burning of the organic part in C(MIM-PTA)_4_. At higher temperatures, the weight loss is due to polyoxometalate decomposition (Fig. 3).

**Fig. 4:**
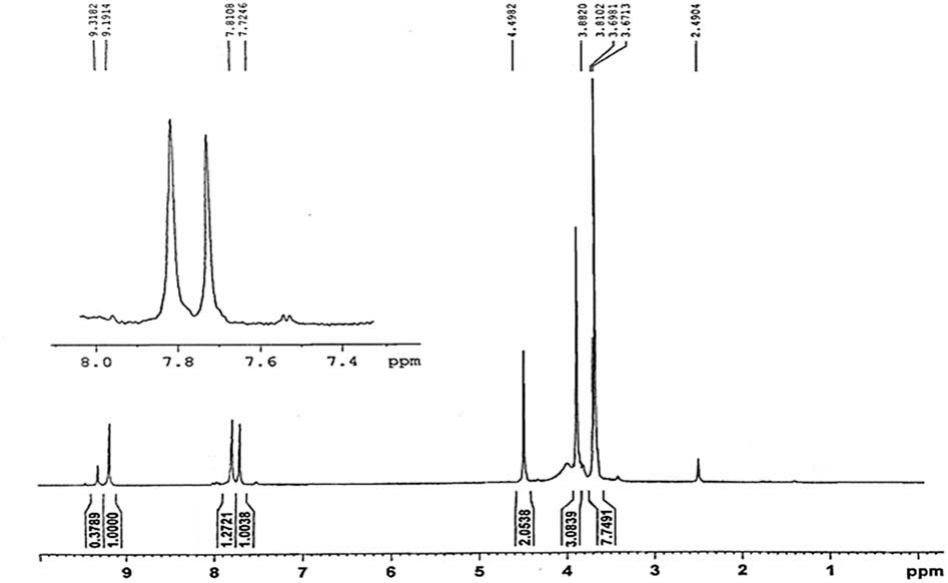
400 MHz ^1^H NMR spectrum in DMSO of pentaerythrityl tetramethylimidazolium bromide.

**Fig. 5:**
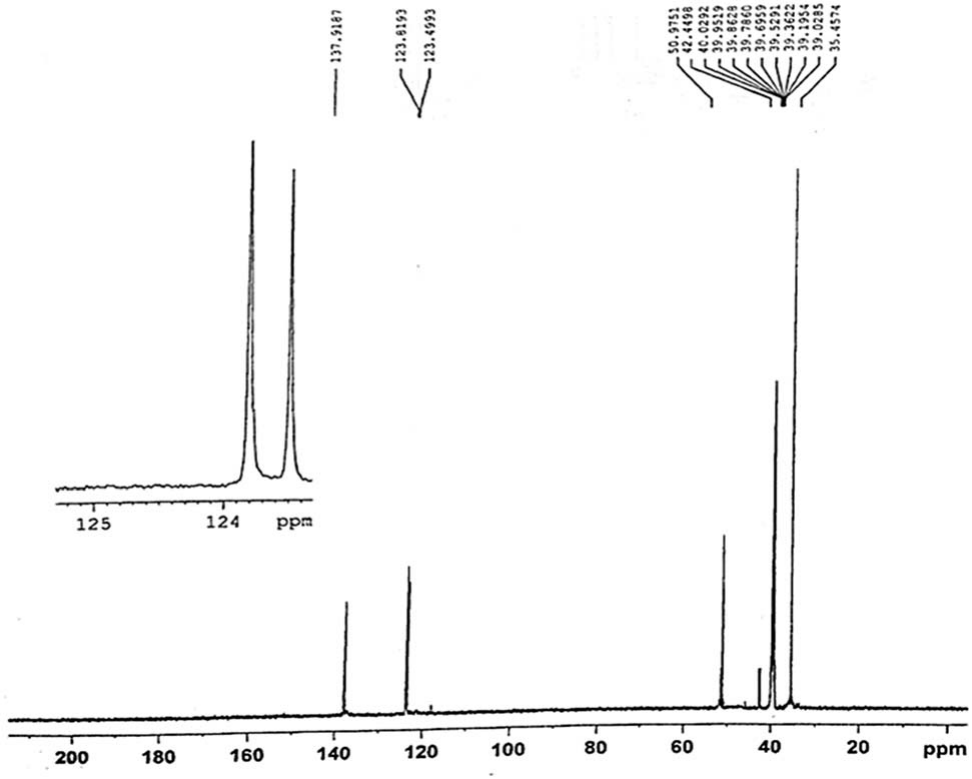
400 MHz ^13^C NMR spectrum in DMSO of pentaerythrityl tetramethylimidazolium bromide.

Fig. 4 and 5 are respectively 400 MHz ^1^H NMR spectrum and ^13^C NMR spectrum in DMSO of pentaerythrityl tetramethylimidazolium bromide. In the ^1^H NMR spectra, the signals of the protons of the methylimidazole ring are respectively *δ* = 3.88, 7.72, 7.81, 9.19 ppm, which are CH_3_, H_1_, H_2_ and H_3_; the signal at *δ* = 4.49 ppm corresponds to the C–CH_2_– protons.

In the ^13^C NMR spectra, the chemical shifts of the carbon in the imidazole ring are respectively *δ* = 123.49,123.81,137.91 ppm, which are C_1_, C_2_ and C_3_; the chemical shift at *δ* = 50.97 ppm corresponds to the CH_2_–N– carbon linking with imidazole ring, which is the C_4_; the chemical shift at *δ* = 42.44 ppm corresponds to the quaternary carbon, which is the C_5_ and CH_3_ the chemical shift at *δ* = 35.45 ppm. So we could confirm that what we synthesized is the target product.

In order to study the pH and the number of free protons, an aqueous solution of NaCl (1 M, 25 mL, pH = 5.9) was mixed with 0.1 g of C(MIM-PTA)_4_. Then, the mixture was magnetically stirred for 24 h, followed by declining the pH to 2.2, which was equal to 1.94 mmol H^+^/g of the catalyst. Comparing C(MIM-PTA)_4_ and the other catalysts, as reported in the literature, for the mentioned reaction revealed.

The advantages of C(MIM-PTA)_4_ over most of them in terms of a higher yield and a shorter reaction time (Table 2). According to these experiences, it can be observed that C(MIM-PTA)_4_ is a highly efficient catalyst useful in the Mannich reaction.

**Table 2:** Comparing the catalytic activity of C(MIM-PTA)_4_ with the reported catalysts in Mannich reaction^a^

Entry	Catalyst	Solvent	Time (min)	Yield (%)	Ref.
1	Pentaerythrityl tetramethyl imidazolium phosphotungstate	H_2_O	60	95	This work
2	Silica sulfuric acid	EtOH	180	96	40
3	HClO_4_–SiO_2_	EtOH	120	98	41
4	Cu (nano particle)	MeOH	540	88	42
5	Zn(OTf)_2_	CH_2_Cl_2_	240	93	43
6	OMMT/polystyrene-SO_3_H	Solvent free	45	95	44
7	ZnO-nanoparticles	H_2_O	10	86	45
8	Heteropoly acid encapsulated SBA-15/TiO_2_	—	360	99	47
9	H_3_PW_12_O_40_	H_2_O	180	84	48
10	HybPOM/HPW_12_	H_2_O	60	96	49
11	[DDPA][HSO_4_]	H_2_O	360	91	50

a Reaction condition: cyclohexanone, benzaldehyde and aniline.

**Fig. 6:**
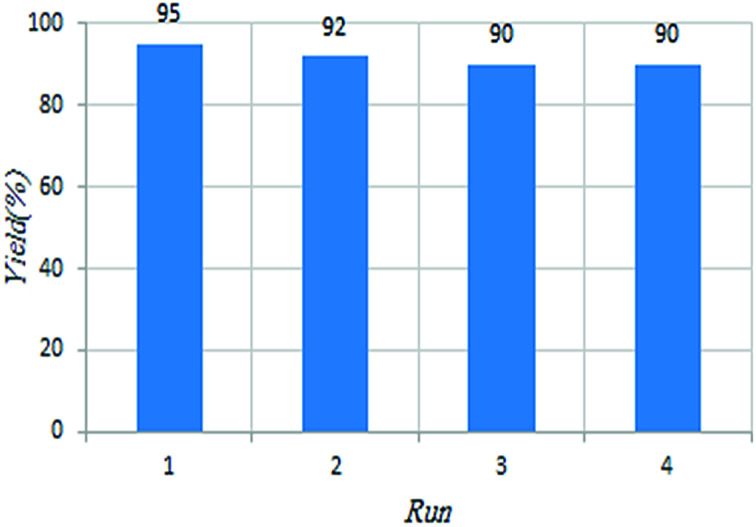
Recyclability of C(MIM-PTA)_4_ in the Mannich reaction.

To demonstrate the superiority C(MIM-PTA)_4_ over the reported catalysts, the reaction of aniline, benzaldehyde and cyclohexanone was considered as a representative example (Fig. 6). It was found that the tetradentate acidic catalyst was chemically stable and reused at least for the next four same reactions with the same catalytic activity. It is different from immobilization of the homogeneous systems, which usually is associated with decreased catalytic activities and potential leaching of catalyst parts.

In view of the leaching problems observed with tungsten supported on the heterogeneous catalyst, quantitative analysis using AAS was employed to determine the amount of metal in the reaction. The heterogeneity of the C(MIM-PTA)_4_ catalyst was examined by carrying out a hot filtration test using aniline, benzaldehyde and cyclohexanone as model substrates. No tungsten could be detected in the liquid phase using AAS and, more significantly, after hot filtration, the reaction of the residual mixture was completely stopped.

## 4. Conclusions

A novel tetradentate acidic catalyst was synthesized and used for the preparation of β-aminocyclohexanones. In this method, pentaerythrityl tetramethyl imidazolium phosphotungstate as a catalyst exhibited much higher catalytic activity and even after four recycles, the product was obtained with high yield and the same purity of the first product. Additionally, the solvent used was water and the product isolation was extremely easy, which is in complete accordance with the green chemistry protocols. The most appealing part of this work is that various cations and anions can be used with widespread capabilities that may be useful in more acid-catalyzed reactions.

## Conflicts of interest

There are no conflicts to declare.

## Supplementary Material

RA-008-C8RA08259F-s001
