# Correction: Designing a novel tetradentate polyoxometalate eco-catalyst for the synthesis of β-aminocyclohexanone derivatives in water

**DOI:** 10.1039/c8ra90101e

**Published:** 2018-12-21

**Authors:** Roya Mozafari, Fariba Heidarizadeh, Maedeh Azaroon

**Affiliations:** Chemistry Department, College of Science, Shahid Chamran University of Ahvaz Ahvaz 61357-4-3169 Iran heidarizadeh@scu.ac.ir +98 6133331042 +98 6135743169

## Abstract

Correction for ‘Designing a novel tetradentate polyoxometalate eco-catalyst for the synthesis of β-aminocyclohexanone derivatives in water’ by Roya Mozafari *et al.*, *RSC Adv.*, 2018, **8**, 40261–40266.

The published affiliation contained an incomplete university name and the corrected version is shown here.

The Royal Society of Chemistry apologises for these errors and any consequent inconvenience to authors and readers.

## Supplementary Material

