# Supplementary material for: Designing a novel tetradentate polyoxometalate eco-catalyst for the synthesis of β-aminocyclohexanone derivatives in water
Source: RSC Adv. 2018 Dec 3;8(70):40261–6. doi: 10.1039/c8ra08259f (PMC9091466; doi:10.1039/c8ra08259f)
Supplement: RA-008-C8RA08259F-s001 [file RA-008-C8RA08259F-s001.pdf]

Electronic Supplementary Material (ESI) for RSC Advances.
This journal is © The Royal Society of Chemistry 2018

Designing a novel tetradentate polyoxometalate eco-catalyst for the synthesis of *β*-aminocyclohexanone derivatives in water

Roya Mozafari[a], Fariba Heidarizadeh*[a],Maedeh Azaroon[a]

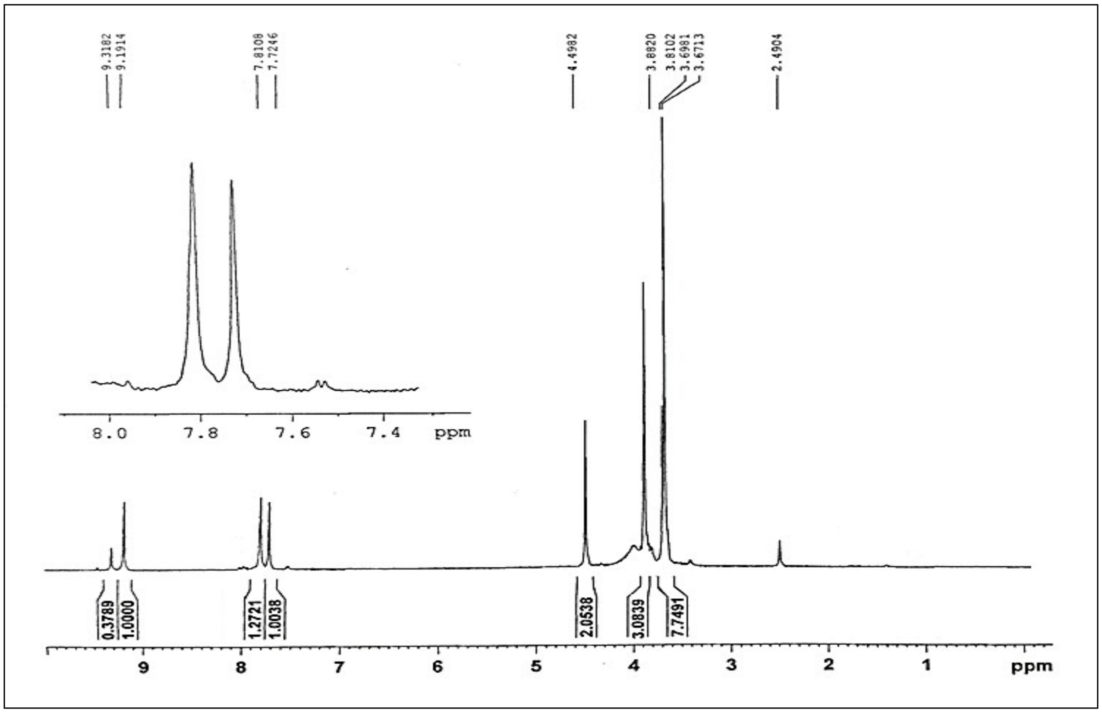

Figure 1. ^{1}H NMR of pentaerythrityl tetramethylimidazolium bromide

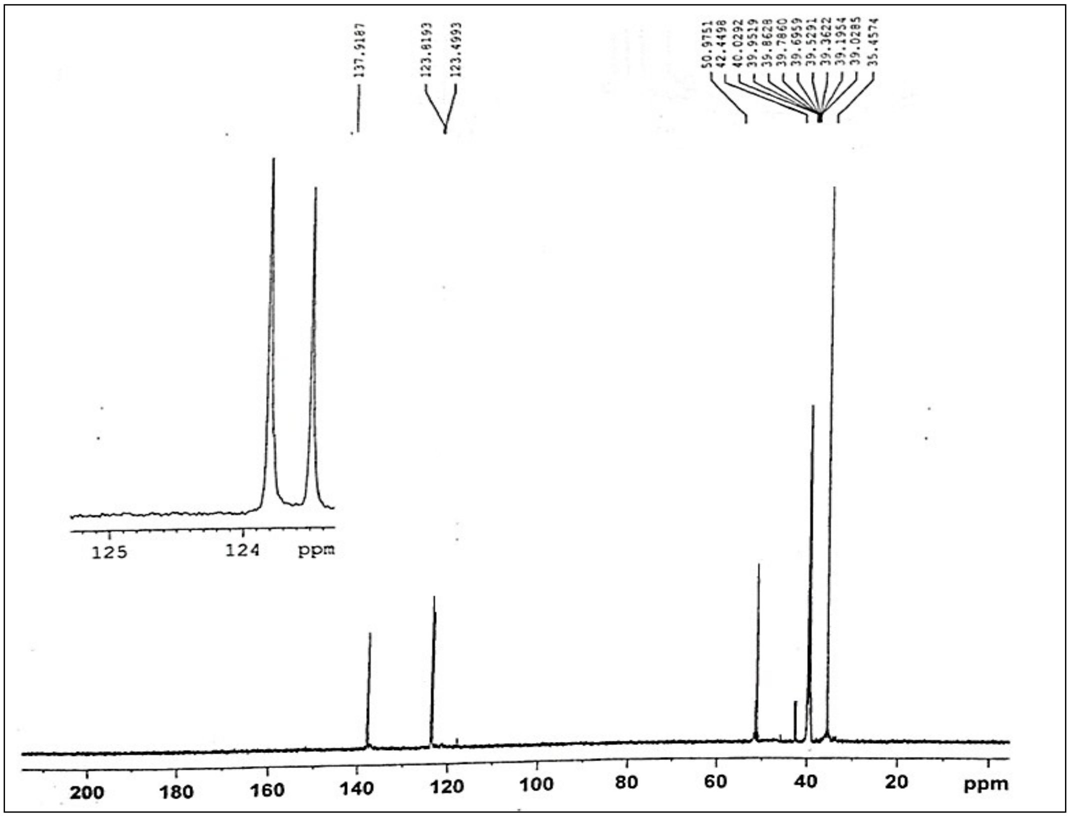

Figure 2. ^{13}C NMR spectrum of pentaerythrityl tetramethylimidazolium bromide

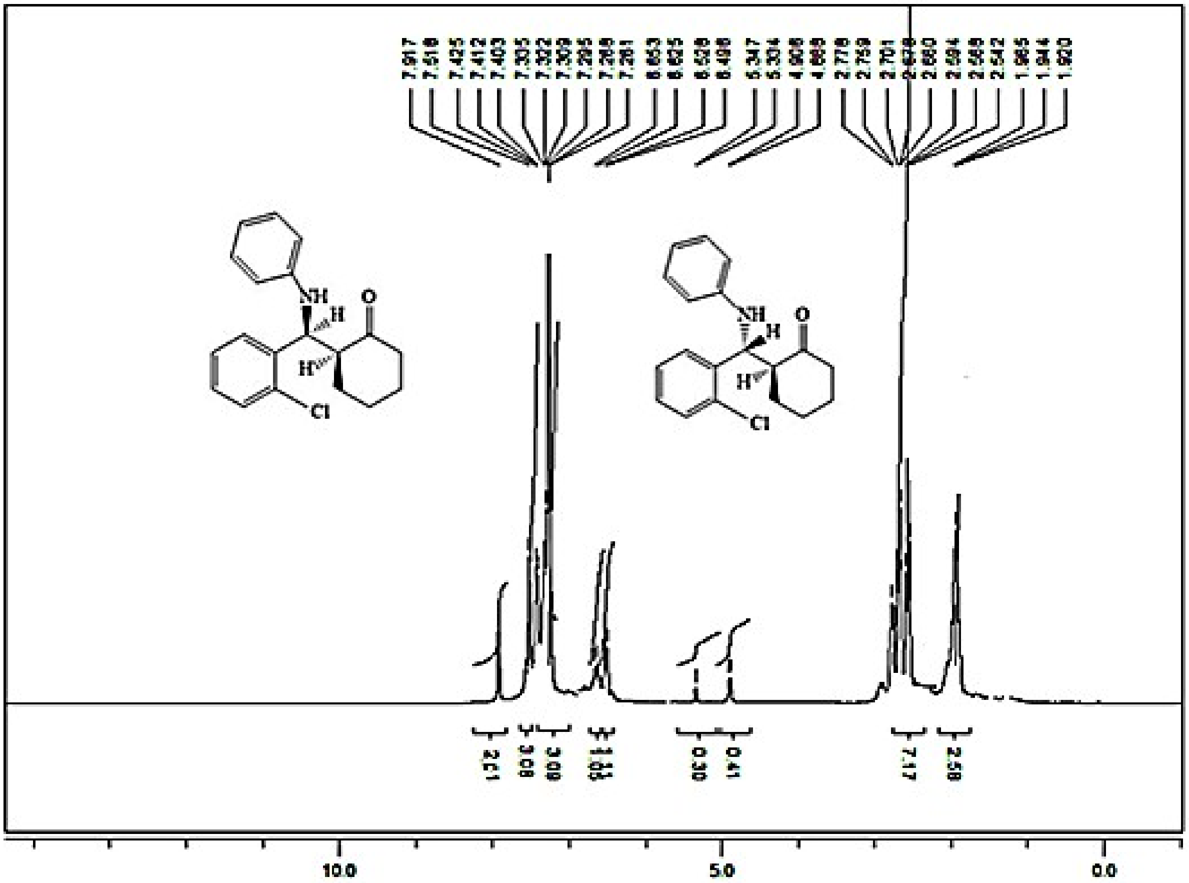

Figure 3. ^{1}H NMR spectrum of 2-((2-chlorophenyl)(phenylamino)methyl)cyclohexanone

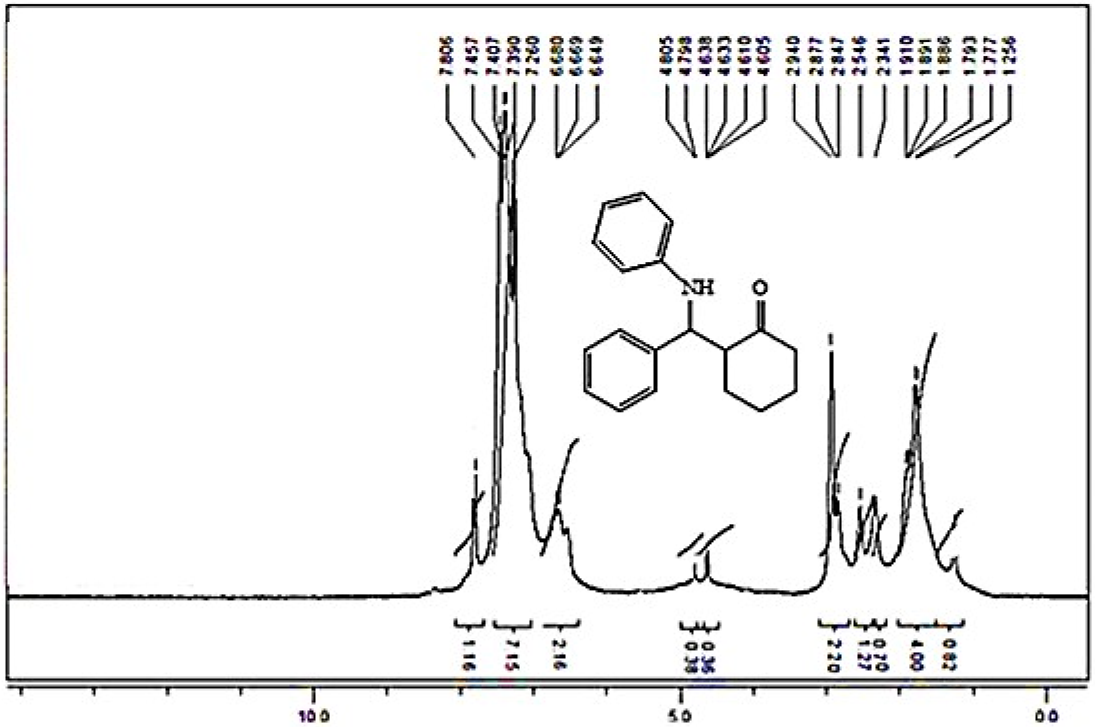

Figure 4. ^{1}H NMR spectrum of 2-(phenyl(phenylamino)methyl)cyclohexanone
